# Supplementary material for: Specific length and structure rather than high thermodynamic stability enable regulatory mRNA stem-loops to pause translation
Source: Nat Commun. 2022 Feb 21;13:988. doi: 10.1038/s41467-022-28600-5 (PMC8861025; doi:10.1038/s41467-022-28600-5)
Supplement: Supplementary file 1 — Supplementary Information [file 41467_2022_28600_MOESM1_ESM.pdf]

Specific length and structure rather than high thermodynamic stability enable regulatory mRNA stem-loops to pause translation

Chen Bao, Mingyi Zhu, Inna Nykonchuk, Hironao Wakabayashi, David H. Mathews and Dmitri N. Ermolenko

Supplementary Information

Supplementary Figures

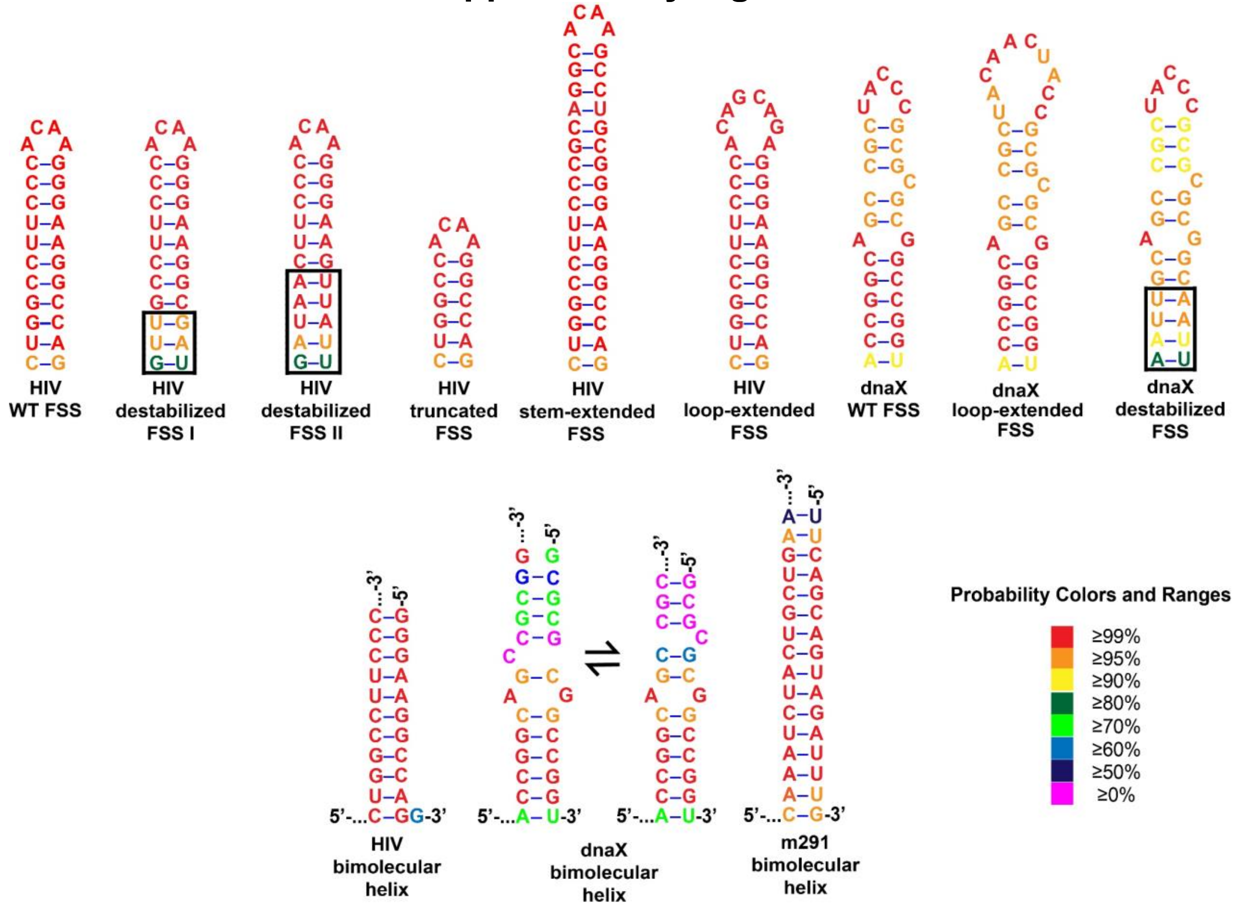

Supplementary Figure 1. Basepair probabilities for model mRNAs (shown in Fig. 1). Maximum expected accuracy structures were calculated using RNAstructure[1], and estimated base pairing probabilities are color annotated. The hairpin stem-loop sequences were mutated to have altered stability, but similarly high pairing probabilities as the wild-type sequences. The dnaX bimolecular helix is predicted to have two structures in equilibrium with folding free energy changes of is -20.3 kcal/mol (left) and -20.2 kcal/mol (right). The conformational difference is whether the C bulges in the mRNA (left) or the trans oligonucleotide (right; more similar to the dnaX hairpin structure). The base of the helix has similarly high probability as the dnaX wild-type hairpin stem-loop.

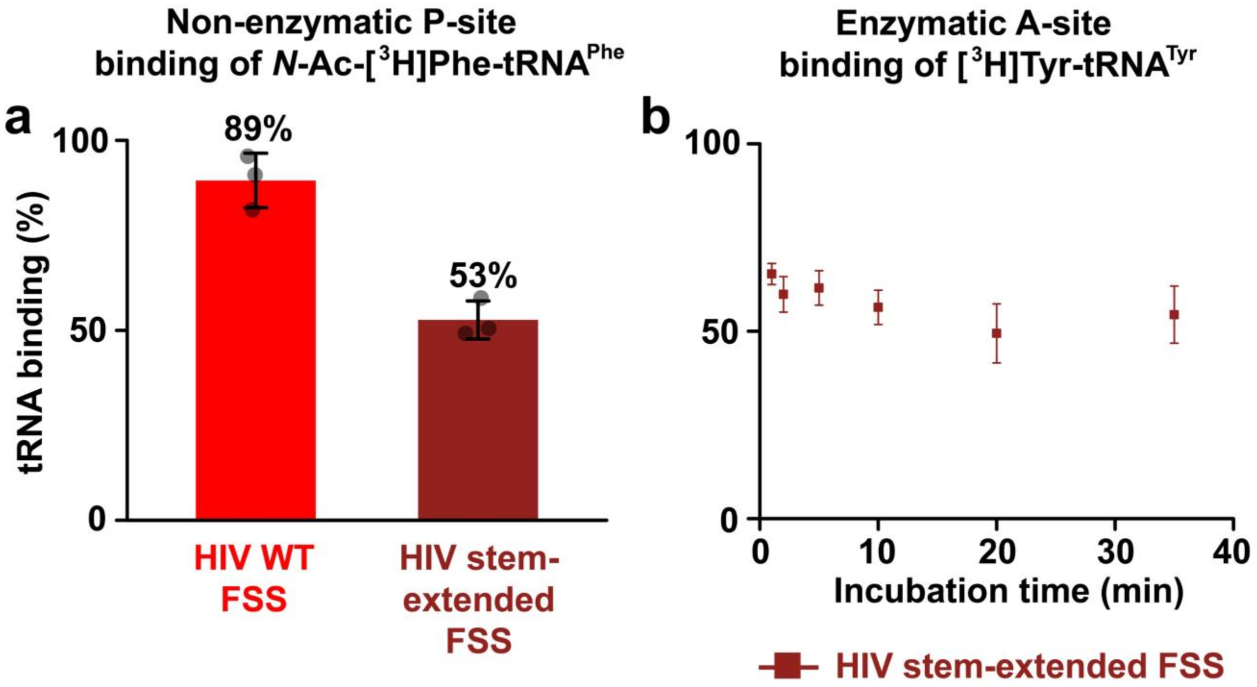

Supplementary Figure 2. Extension of the stem length eliminates the FSS-induced inhibition of tRNA binding. (a) Non-enzymatic binding of *N*-Ac-[^{3}H]Phe-tRNA^{Phe} to the P site of the 70S ribosome programmed with either HIV WT FSS or HIV stem-extended FSS mRNA that was measured by filter-binding assay after 15 minutes of incubation **(Fig 1)**. **(b)** Kinetics of the EF-Tu-catalyzed [^{3}H]Tyr-tRNA^{Tyr} binding to the A site of the 70S ribosome containing P-site *N*-Ac-Met-Phe-tRNA^{Phe}. The binding of radio-labeled tRNA to ribosomes programmed with the FSS-containing mRNA is shown relative to that observed in ribosomes programmed with the HIV ΔFSS mRNA. Data are presented as mean values +/- standard deviations of three independent measurements. Source data are provided as a Source Data file.

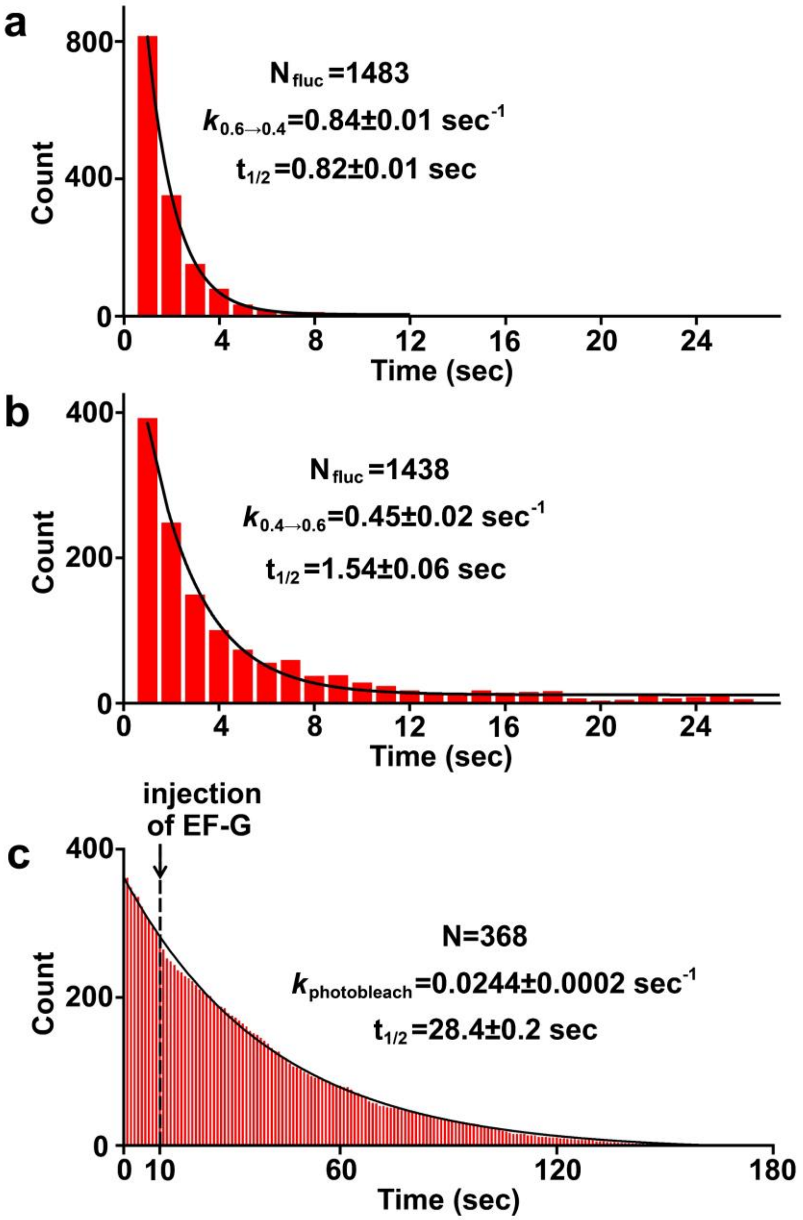

Supplementary Figure 3. Spontaneous fluctuation and photobleaching rates of the S6-cy5/L9-cy3 ribosomes. (a-b) Histograms (1 s binning size) show dwell time of the NR (0.6 FRET) **(a)** and R (0.4 FRET) **(b)** states of the S6/L9-labeled 70S ribosomes containing deacylated $tRNA^{Phe}$ in the P site. Transition rates and half-lives of dwell time were deduced from single exponential fitting shown by black curves. **(c)** Histogram (1 s binning size) show distribution of cy5 life times in translocation smFRET measurements with continues Cy3 excitation in ribosomes programmed with “wild-type” HIV FSS and dnaX FSS mRNAs. Injection of EF-G•GTP is indicated by the arrow. Photobleaching rate and half-life were deduced from single exponential fitting shown by black curve. N indicates the number of FRET traces incorporated into each histogram. Source data are provided as a Source Data file.

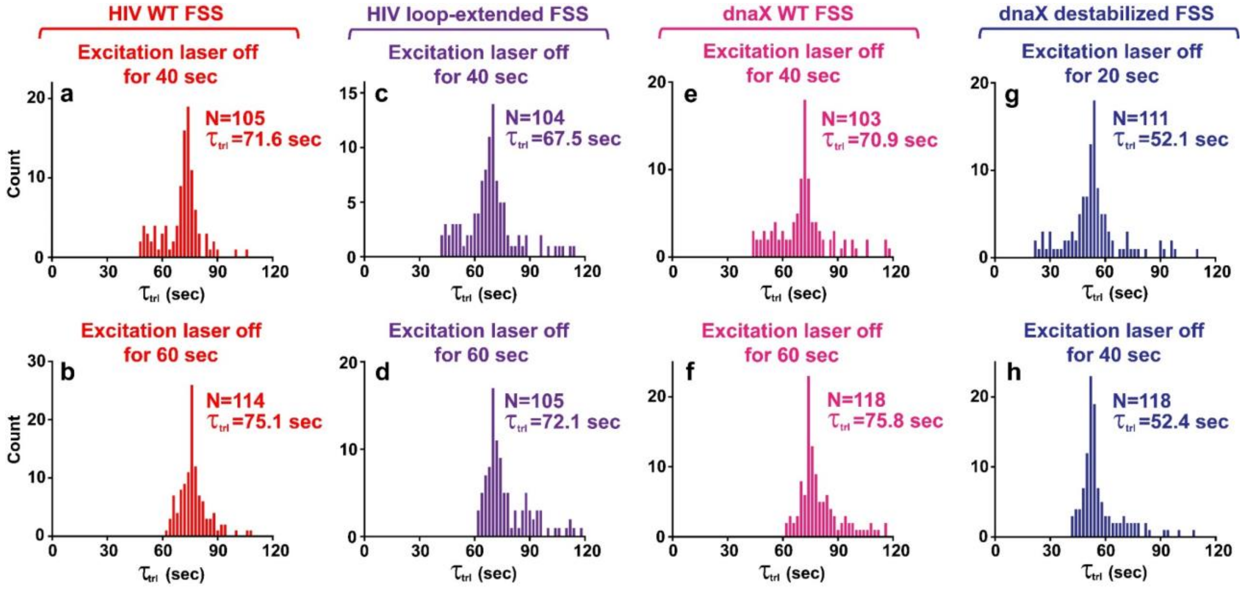

Supplementary Figure 4. Variations of the interval, during which the excitation laser was switched off, did not affect distributions of τ_{trl}. Kinetics of translocation was measured by smFRET experiments with pre-translocation S6-cy5/L9-cy3 ribosomes programmed mRNA containing HIV WT FSS (**a, b**), HIV loop-extended FSS (**c, d**), dnaX WT FSS (**e, f**) or dnaX destabilized FSS (**g, h**), which was positioned 11 nucleotides downstream of P-site codon **(Fig. 1)**. Because these FSS variants strongly inhibited translocation, the excitation laser was turned off after the EF-G injection and switched back on either 20, 40 or 60 s later to extend lifetime of the acceptor fluorophore as indicated. Histograms (2 s binning size) show distributions and median values of τ_{trl}. Source data are provided as a Source Data file.

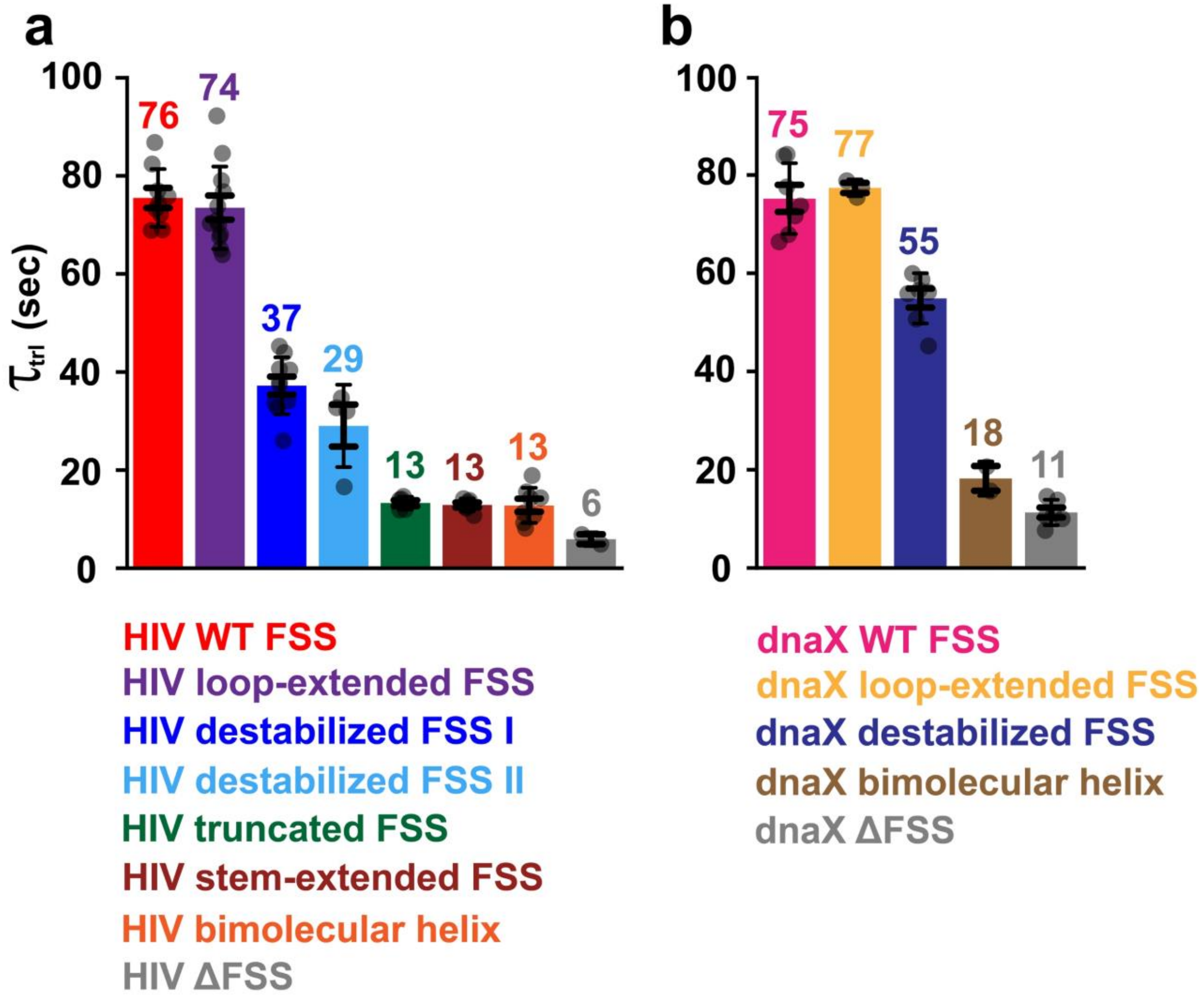

Supplementary Figure 5. The stem length and the presence of the loop are more important for the FSS-induced inhibition of translocation than FSS local and overall thermodynamic stabilities. The S6-cy5/L9-cy3 ribosomes were programmed with mRNA containing either a HIV **(a)** or dnaX **(b)** FSS variants as indicated **(Fig. 1)**. Bar graphs show mean values of τ_{trl} with standard errors of the mean (SEM, thick error bars) and standard deviations (SD, thin error bars) calculated from 2 to 12 independent injection experiments (15-50 traces in each experiments). For all FSS variants but the HIV stem-extended FSS, τ_{trl} represents the dwell time between injection of EF-G•GTP and ribosome translocation in smFRET experiments. For the HIV stem-extended FSS, τ_{trl} corresponds to the dwell time between tRNA binding and translocation **(Fig. 7)**. Source data are provided as a Source Data file.

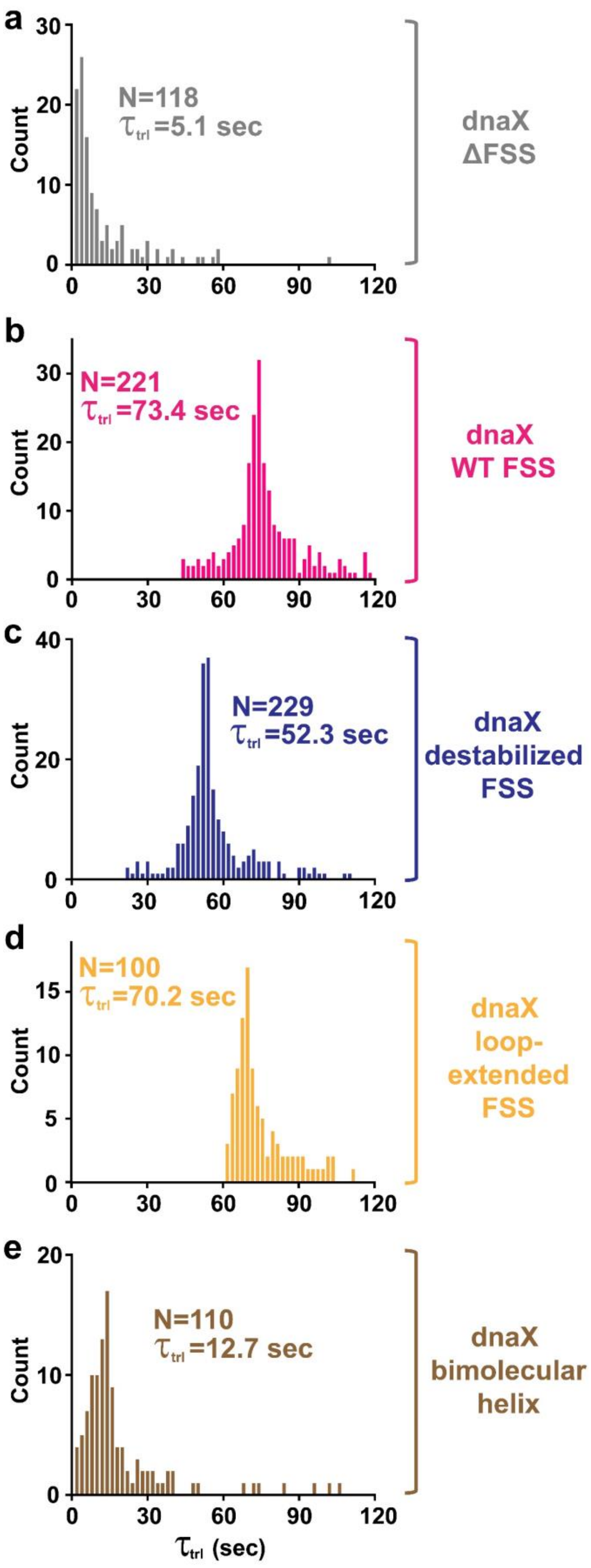

Supplementary Figure 6. The dnaX FSS-induced inhibition of translocation is alleviated by elimination of the loop. Kinetics of translocation was measured by smFRET experiments with pre-translocation S6-cy5/L9-cy3 ribosome programmed with dnaX ΔFSS mRNA **(a)**, dnaX WT FSS mRNA **(b)**, dnaX destabilized FSS mRNA **(c)**, dnaX loop-extended FSS **(d)** or dnaX bimolecular helix mRNA **(e)** **(Fig. 1)**. In each mRNA, the spacer between P-site codon and the downstream secondary structure was 11 nucleotides long. Histograms (2 s binning size) show the distributions and median values of τ_{trl}. N indicates the number of FRET traces incorporated into each histogram. Source data are provided as a Source Data file.

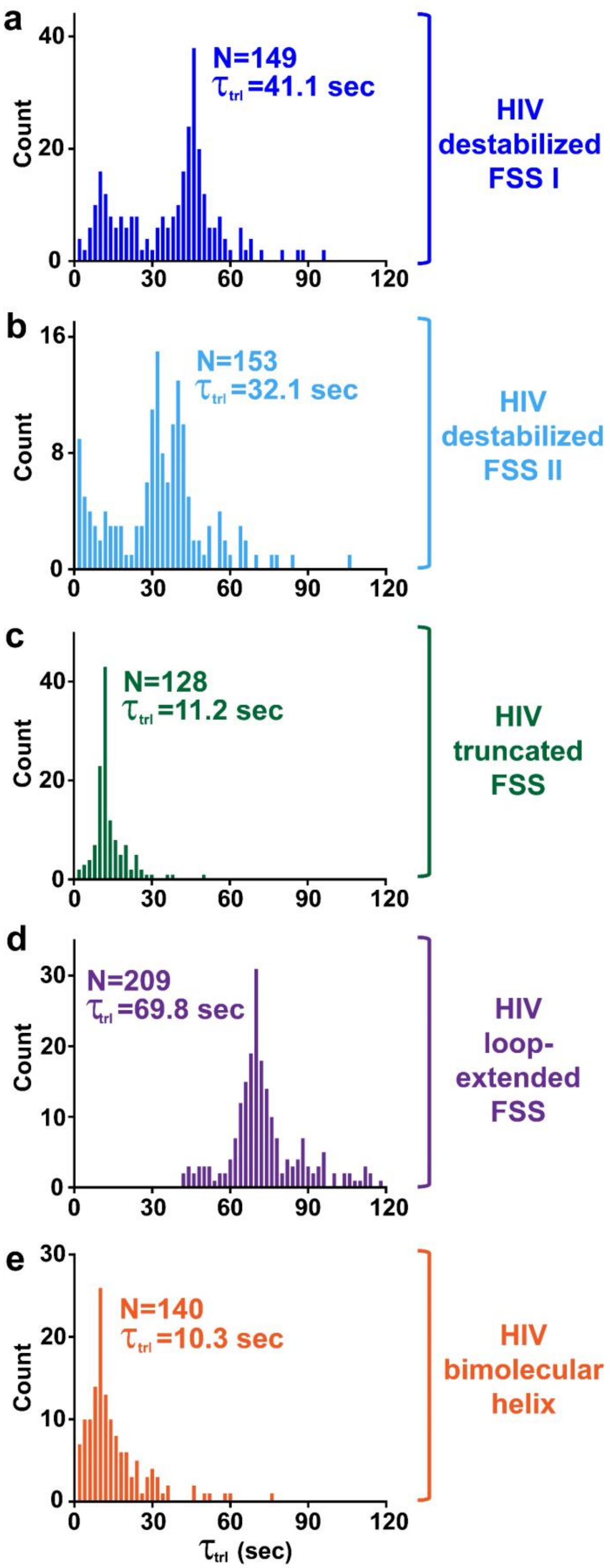

Supplementary Figure 7. The HIV FSS-induced inhibition of translocation is alleviated by truncation of the stem or elimination of the loop. Kinetics of translocation was measured by smFRET experiments (**Fig. 2b**) with pre-translocation S6-cy5/L9-cy3 ribosome programmed with mRNAs containing HIV destabilized FSS I (**a**), HIV destabilized FSS II (**b**), HIV truncated FSS (**c**), HIV loop-extended FSS (**d**) or the bimolecular helix mimicking the native FSS stem (**e**). In each mRNA, the spacer between P-site codon and the downstream secondary structure was 11 nucleotides long. Histograms (2 s binning size) show the distributions and median values of τ_{trl}. N indicates the number of FRET traces included in each histogram. Source data are provided as a Source Data file.

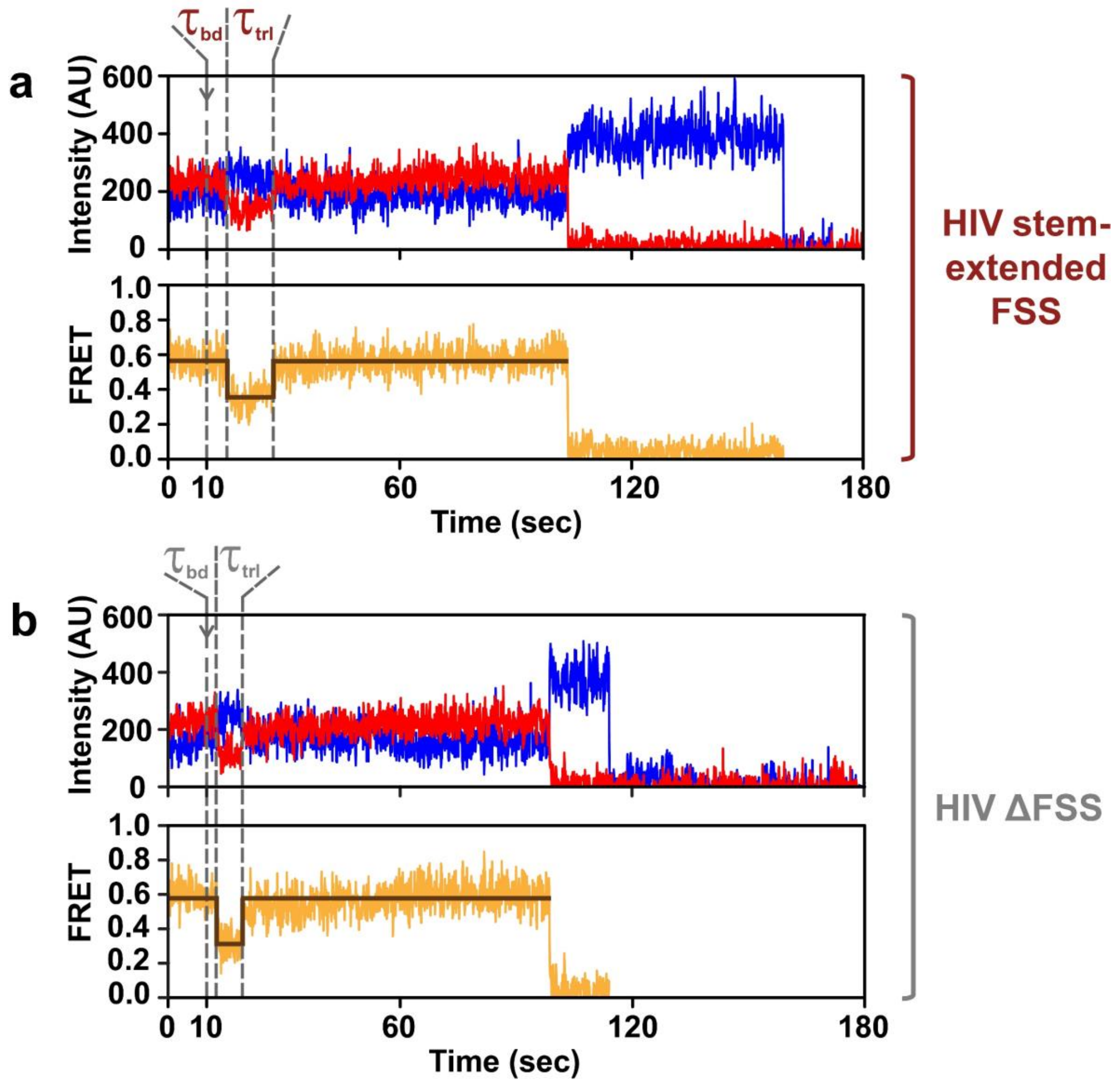

Supplementary Fig 8. Full-length view of FRET traces (a) for Fig. 7 a, and (b) Fig. 7d, that exhibit a single-step photobleaching of both Cy3 and Cy5 fluorophores.

RNA identifier	RNA sequence (5' to 3')
HIV ΔFSS mRNA	GGUUUUUCUUCUGAAGAUAAAGCAACAACAACAAGGCAAGGAGGUAAAAAUGUUCUACAA
HIV **WT FSS** mRNA (11-nt spacer)	GGUUUUUCUUCUGAAGAUAAAGCAACAACAACAAGGCAAGGAGGUAAAAAUGUUCUACAAGAU**CUGGCCUUCCCACAAGGGAAGGCCAG**GGAA
HIV **WT FSS** mRNA (12-nt spacer)	GGUUUUUCUUCUGAAGAUAAAGCAACAACAACAAGGCAAGGAGGUAAAAAUGUUCUACAAGAAU**CUGGCCUUCCCACAAGGGAAGGCCAG**GGAA
HIV **WT FSS** mRNA (13-nt spacer)	GGUUUUUCUUCUGAAGAUAAAGCAACAACAACAAGGCAAGGAGGUAAAAAUGUUCUACGGAAGAU**CUGGCCUUCCCACAAGGGAAGGCCAG**GGAA
HIV **destabilized FSS I** mRNA	GGUUUUUCUUCUGAAGAUAAAGCAACAACAACAAGGCAAGGAGGUAAAAAUGUUCUACAAGAU**GUUGCCUUCCCACAAGGGAAGGCGAU**GGAA
HIV **destabilized FSS II** mRNA	GGUUUUUCUUCUGAAGAUAAAGCAACAACAACAAGGCAAGGAGGUAAAAAUGUUCUACAAGAU**GAUAACUUCCCACAAGGGAAGUUAUU**GGAA
HIV **truncated FSS** mRNA	GGUUUUUCUUCUGAAGAUAAAGCAACAACAACAAGGCAAGGAGGUAAAAAUGUUCUACAAGAU**CUGGCCACAAGGCCAG**GGAA
HIV **stem-extended FSS** mRNA	GGUUUUUCUUCUGAAGAUAAAGCAACAACAACAAGGCAAGGAGGUAAAAAUGUUCUACAAGAU**CUGGCCUUCCCGCAGGCACAAGCCUGCGGGAAGGCCAG**GGAA
HIV **loop-extended FSS** mRNA	GGUUUUUCUUCUGAAGAUAAAGCAACAACAACAAGGCAAGGAGGUAAAAAUGUUCUACAAGAU**CUGGCCUUCCCACAGCAGAGGGAAGGCCAG**GGAA
HIV **bimolecular helix** RNAs	GGUUUUUCUUCUGAAGAUAAAGCAACAACAACAAGGCAAGGAGGUAAAAAUGUUCUACAAGAU**CUGGCCUUCCC**GGAA
	**GGGAAGGCCAGG**
dnaX ΔFSS mRNA	GGUUUUUCUUCUGAAGAUAAAGCAACAACAACAAGGCAAAGGGAGCAACCAUGGUAUUCUACAGAGAACCGG
dnaX_Slip ΔFSS mRNA	GGUUUUUCUUCUGAAGAUAAAGCAACAACAACAAGGCAAAGGGAGCAACCAUGGUAAAAAAGAGAGAACCGG
dnaX **WT FSS** mRNA	GGUUUUUCUUCUGAAGAUAAAGCAACAACAACAAGGCAAAGGGAGCAACCAUGGUAUUCUACAGAGA**ACCGGCAGCCGCUACCCGCGCGCGGCCGGU**GAAUAACGGGAUC
dnaX_Slip **WT FSS** mRNA	GGUUUUUCUUCUGAAGAUAAAGCAACAACAACAAGGCAAAGGGAGCAACCAUGGUAAAAAAGAGAGA**ACCGGCAGCCGCUACCCGCGCGCGGCCGGU**GAAUAACGGGAUC
dnaX **destabilized FSS** mRNA	GGUUUUUCUUCUGAAGAUAAAGCAACAACAACAAGGCAAAGGGAGCAACCAUGGUAUUCUACAGAGA**AAUUGCAGCCGCUACCCGCGCGCGGCAAUU**GAAUAACGGGAUC
dnaX_Slip **destabilized FSS** mRNA	GGUUUUUCUUCUGAAGAUAAAGCAACAACAACAAGGCAAAGGGAGCAACCAUGGUAAAAAAGAGAGA**AAUUGCAGCCGCUACCCGCGCGCGGCAAUU**GAAUAACGGGAUC

dnaX loop-extended FSS mRNA	**GGUUUUUCUUCUGAAGAUAAAG**CAACAACAACAAGGCAAAGGGAGCAACCAUGGUAUUCUACAGAGAACCGGCAGCCGCUACAACUACCGCGCGCGGCCGGUGAAUAACGGGAUC
dnaX bimolecular helix RNAs	**GGUUUUUCUUCUGAAGAUAAAG**CAACAACAACAAGGCAAAGGGAGCAACCAUGGUAUUCUACAGAGAACCGGCAGCCGCGGAUC
	GCGCGCGGCCGGU
dnaX_Slip bimolecular helix RNAs	**GGUUUUUCUUCUGAAGAUAAAG**CAACAACAACAAGGCAAAGGGAGCAACCAUGGUAAAAAAGAGAGAACCGGCAGCCGCGGAUC
	GCGCGCGGCCGGU
m291 bimolecular helix RNAs	GUAAAGUGUCAUAGCACCAACUGUUAAUUAAAUUAAAUUAAAAAGGAAAUAAAAAUGUUUGUAUACAAAUCUACUGCUGAACUCGCUGCACAAAUGGCUAAACUGAAUGGCAAUAAA**GGUUUUUCUUCUGAAGAUAAAG**
	UUCAGCAGUAGAUUUG

Supplementary table 1. RNA sequences

The RNA sequences are shown in 5'-to-3' direction. In each RNA, the Shine-Dalgarno-like sequence and the RNA spacer extending from the first nucleotide of the P-site codon to the 5' end of the FSS are colored green and highlighted, respectively. The native dnaX slippery sequence is colored pink in dnaX_Slip mRNAs. The **sequences of the FSS variants and the biomolecular helices** are color coded as in Figure 1. Every model mRNA contains a **handle sequence** complementary to the biotinylated DNA oligo, which is used to immobilize the mRNA in smFRET experiments (bold and underlined).

Supplementary table 2. The HIV and dnaX bimolecular helices formed by annealing two complementary RNA strands.

	ΔG (kcal/mol)	K_{eq}	k_{off} (s^{-1})	$t_{1/2}$ (s)	[Input] (μM)	[Free RNA] (μM)	[bimolecular helix] (μM)
HIV bimolecular helix (310.15 K)	-27.8	3.90×10^{19}	2.56×10^{-14}	2.70×10^{13}	0.60	1.24×10^{-7}	0.60
HIV bimolecular helix (293.15 K)	-34.8	4.27×10^{28}	2.34×10^{-23}	2.96×10^{22}	0.30	2.65×10^{-12}	0.30
dnaX bimolecular helix (310.15 K)	-28.6	1.43×10^{20}	7.00×10^{-15}	9.90×10^{13}	0.60	6.48×10^{-8}	0.60
dnaX bimolecular helix (293.15 K)	-39.7	3.99×10^{29}	2.51×10^{-24}	7.66×10^{19}	0.30	8.68×10^{-13}	0.30

In accordance with the incubation temperatures used in filter-binding and smFRET experiments, free energy change ΔG° for RNA annealing were calculated at 37 °C (310.15 K) or at room temperature (293.15 K), respectively. Equilibrium constants K_{eq} were calculated from $K_{eq}=\exp(-\Delta G/RT)$, where temperature T was either 310.15 or 293.15 K. $R=1.987\times10^{3}$ $kcal\cdot K^{-1}\cdot mol^{-1}$. Dissociation rates k_{off} were calculated using equation $K_{eq}=k_{on}/k_{off}$, where association rate k_{on} was approximated to 10^{6} s^{-1} as its value is less temperature sensitive[4]. Half-life $t_{1/2}$ of each bimolecular helix were calculated from $t_{1/2}=\ln(2)/k_{off}$. In the calculation, prior to annealing, initial concentrations of the two complementary RNA strands [input] were equal (0.6 or 0.3 μM). Thus, concentrations of free and annealed RNAs, [free RNA] and [bimolecular helix], were determined by solving equations [bimolecular helix]+2×[free RNA]=2×[input] and K_{eq}=[bimolecular helix]/[free RNA]^{2}.

Supplementary table 3. Specific length and structure of the FSS significantly affect the FSS-induced inhibition of A-site tRNA binding

	k ***(min^{-1})***	**standard deviation (min^{-1})**	**p value**
**HIV WT FSS**	**0.15**	**0.04**	
**HIV destabilized FSS I**	**0.16**	**0.02**	**0.85**
**HIV destabilized FSS II**	**0.24**	**0.04**	**0.15**
**HIV truncated FSS**	**0.9**	**0.1**	**0.0002**
**HIV loop-extended FSS**	**0.35**	**0.06**	**0.03**
**dnaX WT FSS**	**0.26**	**0.08**	
**dnaX destabilized FSS**	**0.33**	**0.04**	**0.46**
**HIV loop-extended FSS**	**0.25**	**0.05**	**0.91**

Pseudo-first-order rate constants (*k*) of A-site Tyr-tRNA^{Tyr} binding measured in the presence of HIV or dnaX FSS mRNA variants by filter-binding assay (**Fig. 3**). p values determined by two-tailed Student t-test colored in red indicate that the rate of tRNA binding in the presence of these mRNA variants is different from binding observed in the presence of the wild-type mRNA variant. Standard deviations are calculated from 3 to 4 independent measurements.

References:

1. Reuter, J.S. & Mathews, D.H. RNAstructure: software for RNA secondary structure prediction and analysis. *BMC Bioinformatics* **11**, 129 (2010).
2. Larsen, B., Gesteland, R.F. & Atkins, J.F. Structural probing and mutagenic analysis of the stem-loop required for Escherichia coli dnaX ribosomal frameshifting: programmed efficiency of 50%. *J Mol Biol* **271**, 47-60 (1997).
3. Larsen, B., Wills, N.M., Gesteland, R.F. & Atkins, J.F. rRNA-mRNA base pairing stimulates a programmed -1 ribosomal frameshift. *J Bacteriol* **176**, 6842-51 (1994).
4. Bloomfield, V.A., Crothers, D.M. & Tinoco, I. *Nucleic acids : structures, properties, and functions*, x, 794 p. (University Science Books, Sausalito, Calif., 2000).
