# Supplementary material for: Specific length and structure rather than high thermodynamic stability enable regulatory mRNA stem-loops to pause translation
Source: Nat Commun. 2022 Feb 21;13:988. doi: 10.1038/s41467-022-28600-5 (PMC8861025; doi:10.1038/s41467-022-28600-5)
Supplement: Supplementary file 3 — Reporting Summary [file 41467_2022_28600_MOESM3_ESM.pdf]

nature research

Corresponding author(s): Dmitri N. Ermolenko

Last updated by author(s): Jan 25, 2022

Reporting Summary

Nature Research wishes to improve the reproducibility of the work that we publish. This form provides structure for consistency and transparency in reporting. For further information on Nature Research policies, see our Editorial Policies and the Editorial Policy Checklist.

Statistics

For all statistical analyses, confirm that the following items are present in the figure legend, table legend, main text, or Methods section.

n/a	Confirmed	
[ ]	[x]	The exact sample size (*n*) for each experimental group/condition, given as a discrete number and unit of measurement
[ ]	[x]	A statement on whether measurements were taken from distinct samples or whether the same sample was measured repeatedly
[ ]	[x]	The statistical test(s) used AND whether they are one- or two-sided<br>*Only common tests should be described solely by name; describe more complex techniques in the Methods section.*
[x]	[ ]	A description of all covariates tested
[x]	[ ]	A description of any assumptions or corrections, such as tests of normality and adjustment for multiple comparisons
[ ]	[x]	A full description of the statistical parameters including central tendency (e.g. means) or other basic estimates (e.g. regression coefficient) AND variation (e.g. standard deviation) or associated estimates of uncertainty (e.g. confidence intervals)
[ ]	[x]	For null hypothesis testing, the test statistic (e.g. *F*, *t*, *r*) with confidence intervals, effect sizes, degrees of freedom and *P* value noted<br>*Give P values as exact values whenever suitable.*
[x]	[ ]	For Bayesian analysis, information on the choice of priors and Markov chain Monte Carlo settings
[x]	[ ]	For hierarchical and complex designs, identification of the appropriate level for tests and full reporting of outcomes
[x]	[ ]	Estimates of effect sizes (e.g. Cohen's *d*, Pearson's *r*), indicating how they were calculated

Our web collection on statistics for biologists contains articles on many of the points above.

Software and code

Policy information about availability of computer code

Data collection	smFRET data were collected using Single software, version 0.4 (freely available from Taekjip Ha's laboratory at http://ha.med.jhmi.edu/resources/)
Data analysis	RNAstructure (version 6.2) was used to prediction the RNA secondary structure and thermodynamic stability of corresponding structures (https://rna.urmc.rochester.edu/RNAstructure.html). smFRET data were analyzed using Matlab scripts and HaMMy software, version 1.0.0 (freely available from Taekjip Ha's laboratory at http://ha.med.jhmi.edu/resources/).

For manuscripts utilizing custom algorithms or software that are central to the research but not yet described in published literature, software must be made available to editors and reviewers. We strongly encourage code deposition in a community repository (e.g. GitHub). See the Nature Research guidelines for submitting code & software for further information.

Data

Policy information about availability of data

All manuscripts must include a data availability statement. This statement should provide the following information, where applicable:
- Accession codes, unique identifiers, or web links for publicly available datasets
- A list of figures that have associated raw data
- A description of any restrictions on data availability

Except for the values of fluorescence intensity in smFRET measurements, all other data supporting the findings of this study have been presented within this article and source data file provided with this paper. Due to the lack of a public repository for smFRET traces, the measured fluorescence intensities evolving over imaging time are available from the corresponding authors upon request.

Field-specific reporting

Please select the one below that is the best fit for your research. If you are not sure, read the appropriate sections before making your selection.

☒ Life sciences ☐ Behavioural & social sciences ☐ Ecological, evolutionary & environmental sciences

For a reference copy of the document with all sections, see nature.com/documents/nr-reporting-summary-flat.pdf

Life sciences study design

All studies must disclose on these points even when the disclosure is negative.

Sample size	All smFRET and biochemical data were obtained from at least three independent experiments according to practices standard for biochemical experiments. For kinetic analysis, at least 100 smFRET traces were used in each smFRET experiments. With these samples sizes, SD of the mean was less than 20% of mean value (in the vast majority of experiments, SD was less than 10% of mean value).
Data exclusions	As stated in Methods, in TIRF/smFRET experiments, fluorescence vs time traces were excluded if they did not show single-step Cy3 and Cy5 photobleaching or anti-correlated changes in Cy3 and Cy5 fluorescence. This was done to assure that only actual smFRET traces were analyzed while spurious fluorescent signals, signals from ribosomes lacking fluorecsent Cy5 and fluorescent signals from ribosome aggregates were excluded. To determine the rate of translocation, smFRET traces, which did not show a transition from 0.4 to stable (i.e. lasting over 4s) 0.6 FRET state after EF-G injection, were excluded to assure that only bone fide translocation transitions were used in subsequent kinetic analysis.
Replication	All smFRET and biochemical data were obtained from at least three independent experiments. All observations made in these experiments were reproducible.
Randomization	N/A. No subject allocation/selection for a specific treatment was involved in experimental design (subjects of experimental treatments were ribosomes, i.e. macromolecule complexes). In other words, in each experiment, all ribosomes in the sample were subjected to the same experimental treatment. No human subjects were involved in research.
Blinding	N/A. No human subjects were involved in research.

Reporting for specific materials, systems and methods

We require information from authors about some types of materials, experimental systems and methods used in many studies. Here, indicate whether each material, system or method listed is relevant to your study. If you are not sure if a list item applies to your research, read the appropriate section before selecting a response.

Materials & experimental systems

n/a	Involved in the study	
☒	☐	Antibodies
☒	☐	Eukaryotic cell lines
☒	☐	Palaeontology and archaeology
☒	☐	Animals and other organisms
☒	☐	Human research participants
☒	☐	Clinical data
☒	☐	Dual use research of concern

Methods

n/a	Involved in the study	
☒	☐	ChIP-seq
☒	☐	Flow cytometry
☒	☐	MRI-based neuroimaging
